# The Early Development Instrument: an evaluation of its five domains using Rasch analysis

**DOI:** 10.1186/s12887-016-0543-8

**Published:** 2016-01-20

**Authors:** Margaret Curtin, John Browne, Anthony Staines, Ivan J. Perry

**Affiliations:** Department of Epidemiology and Public Health, University College Cork, Floor 4, Western Gateway Building, Cork, Ireland; School of Nursing and Human Sciences, Dublin City University, Dublin, Ireland

## Abstract

**Background:**

Early childhood development is a multifaceted construct encompassing physical, social, emotional and intellectual competencies. The Early Development Instrument (EDI) is a population-level measure of five domains of early childhood development on which extensive psychometric testing has been conducted using traditional methods. This study builds on previous psychometric analysis by providing the first large-scale Rasch analysis of the EDI. The aim of the study was to perform a definitive analysis of the psychometric properties of the EDI domains within the Rasch paradigm.

**Methods:**

Data from a large EDI study conducted in a major Irish urban centre were used for the analysis. The unidimensional Rasch model was used to examine whether the EDI scales met the measurement requirement of invariance, allowing responses to be summated across items. Differential item functioning for gender was also analysed.

**Results:**

Data were available for 1344 children. All scales apart from the Physical Health and Well-Being scale reliably discriminated between children of different levels of ability. However, all the scales also had some misfitting items and problems with measuring higher levels of ability.

Differential item functioning for gender was particularly evident in the emotional maturity scale with almost one-third of items (9 out of 30) on this scale biased in favour of girls.

**Conclusion:**

The study points to a number of areas where the EDI could be improved.

## Background

Early childhood development is a key indicator of future health and well-being [1]. It is a multifaceted construct encompassing physical, social, emotional and intellectual competencies. In the early years, child development is synonymous with child health, which can be defined as the extent to which children realise their full developmental potential [2].

From a population health perspective early childhood development is both an indicator of child health outcomes and a predictor of future health problems [3]. When compared to adult health it is also very susceptible to environmental influences. It is a dynamic process which changes rapidly over time, particularly between gestation and six years of age. As a result, measurement of early childhood development has to be age-specific and multi-dimensional [4].

The majority of measures of early childhood development have been designed by psychologists or educationalists and are clinically-based diagnostic tools, with the intention of determining whether an individual child has a disability or underlying condition [5]. A potentially greater burden of risk lies with the substantially larger number of children with less pronounced developmental delay [6]. In this context, a population-level approach which can measure the developmental health of children across the spectrum is required.

The Early Development Instrument (EDI) is a population-level measure designed at the Offord Centre for Child Studies, McMaster University, Hamilton, Ontario to measure the extent to which children have attained the physical, social, emotional and cognitive maturity necessary to engage in school activities [7]. The EDI is a community or population level measure, not an individual screening or diagnostic tool. The EDI follows a population model for health improvement: small modifications of risk for large numbers are more effective at producing change than large modifications for small numbers [8]. It can be retrospective, focusing on early childhood development outcomes; or predictive, informing school and child-health programmes [7]. It is based on a broad conceptualisation of school readiness which goes beyond language and cognitive ability to include the extent to which the child has gained the developmental maturity (physically, socially and emotionally, as well as cognitively) to engage in and benefit from school activities [9]. Children who score in the lowest 10 % of the study population in one or more of the five domains of the EDI are classed as ‘vulnerable’. The 10 % cut-off has been recommended because it is usually higher than clinical cut-off points and should therefore include children who may be more difficult to diagnose [10].

The EDI is an internationally recognised measure of early childhood development at school entry age [11]. It has been used in 24 countries worldwide. In Australia, where it was administered as the Australian Early Development Index (AEDI) until 2014 when it became the Australian Early Development Census (AEDC), total population coverage has been achieved. Near-total population coverage has been reached in Canada. Its utility in informing regional and national policy on early childhood care and education and in tracking changes in child development outcomes over time is well recognised [12].

Extensive psychometric testing has been completed on the EDI in Canada and Australia [7]. It has high internal consistency with Cronbach’s alpha coefficients of between 0.84 and 0.96 for the five domains [9]. In the current Cork study the EDI was shown to have similar internal consistency with Cronbach’s alpha coefficients of between 0.8 and 0.96 [11]. In Australia, the AEDI was implemented alongside the Longitudinal Study of Australian Children (LSAC) in a subset of the population allowing for correlation with other teacher and parental administered instruments. Results showed strong correlations between the AEDI and other teacher-rated measures. However, correlations with parent-rated measures were weak [13]. Factor analysis was conducted on data from Canada, Australia, Jamaica and Washington State with items loading on to the correct factors across all countries [14]. In a further study of 26,005 children in British Columbia, confirmatory factor analysis was used to demonstrate the unidimensionality of each domain [15]. In examining the predictive validity of the EDI to fourth grade, D’Anguilli et al. [16] found that children who were vulnerable (i.e. in the lowest 10 % of the population in one or more domains of the EDI) in the first year of education were two to four times more likely to score below expectations in Grade 4. There was a linear increase in the risk of scoring below expectations with vulnerability in additional domains. Two studies examined the performance of the EDI across diverse populations and concluded that the EDI was fair and unbiased across gender, language and aboriginal status [6, 17].

There is also some evidence questioning the validity of the EDI. Although correlations between the EDI language and cognitive development domains and the Peabody Picture Vocabulary Test (PPVT) showed similar levels of correlation across four countries, the results showed that low scores in the this domain did not indicate a high probability that a child would have a language problem [14]. A further study, conducted in Canada, comparing the EDI with four directly administered tests of school readiness found significant correlations at the level of the overall instrument but not at the domain level [18].

All the psychometric tests outlined above were conducted using traditional psychometric methods based upon Classical Test Theory (CTT). Only two studies have been conducted using more modern psychometric techniques. In 2004 a Rasch analysis of the EDI was conducted prior to its adaptation for use in Australia as the AEDI. That analysis showed the EDI had generally adequate scale properties within the Rasch paradigm but had disordered thresholds on all items with five response options [19]. The EDI was subsequently adjusted to include only two and three item responses – this was the version used in the Irish study. A subsequent Rasch analysis of the new scales was conducted in a small sample of 116 children in Sweden [20]. This study took the approach of removing misfitting items, after which, all scales except physical health and well-being functioned well. However, the study had too low a sample size to perform a definitive analysis and should be considered an exploratory study [21].

This study builds on previous psychometric analysis by providing the first large-scale Rasch analysis of the current version of the EDI. Data from a large study conducted in a major Irish urban centre were used for the analysis [11].

## Methods

A cross-sectional study of child development was carried out with children in their first year of formal education in 42 of the 47 primary schools in Cork City and a further five schools in an adjoining rural area in 2011. The five city schools which declined to take part in the study were representative of a cross-section of schools in the study area - one boys’ school, one girls’ school, one large mixed, middle income school, one designated disadvantaged school and one Irish-speaking school – and their omission would not have affected the representativeness of the demographic composition of the study.

All eligible children in the participating schools were invited to be included in the study. Eligibility criteria were: being in the latter half of the first year of formal education (i.e. having completed minimum of 4 to 5 months of education), being known by the teacher for more than 1 month and not having left the school.

Strengthening the Reporting of Observational studies in Epidemiology (STROBE) guidelines were adhered to in developing the study and a STROBE checklist compiled.

### Data collection

The EDI is a teacher-completed questionnaire based on five months’ observation of the children from the date when they start school. In the current study it was administered in the latter half of the first year of formal education. The teachers in this study were given a short period of training on the administration of the EDI and were each issued with an EDI guide book. Children were not present when the questionnaire was completed and no individual identifiers were recorded. Each child was assigned a unique identifier which was used on the questionnaire.

### Ethical considerations

Passive consent was used in line with previous EDI studies in Canada. A total of seven parents opted not to participate. Ethical approval was granted by the Clinical Research Ethics Committee of the Cork Teaching Hospitals by whom the opt out consent mechanism was reviewed and approved.

### The Early Development Instrument: structure and scoring

The EDI consists of five domains or scales, made up of 104 questions. The domains are:

- **Physical Health and Well-Being (PHWB).** (13 questions) Physical independence, appropriate clothes and nutrition, fine and gross motor skills
- **Social Competence (SC).** (26 questions) Self-confidence, ability to play, get on with others and share
- **Emotional Maturity (EM).** (30 questions) Ability to concentrate, help others, age appropriate behaviours
- **Language and Cognitive Development (LCD).** (26 questions) Interest in reading and writing, can count and recognise numbers, shapes
- **Communication Skills and General Knowledge (CSGK).** (8 questions) Can communicate with adults and children has an appropriate knowledge of the world.

The physical health and well-being scale has 13 items. Seven items have two response options, scored 0 and 1, and six items have three response options, scored 0, 1 and 2. The social competence scale has 26 items, the emotional maturity scale has 30 items and the communication and general knowledge scale has 8 items. All items on these three scales have three response options, scored 0, 1 and 2. The language and cognitive development scale has 26 items all of which have two response options, scored 0 and 1. Lower scores on all items for all scales represent lower levels of the latent trait being measured.

### Analysis

#### The Rasch model

The Rasch model takes its name from the Danish mathematician Georg Rasch and refers to a group of statistical techniques used as a mathematical approach to assessing measurement scales [22]. The model assumes that the probability of a person responding in a certain way to an item on a psychometric scale is a logistic function of the difference between that person’s ability and the individual item’s difficulty [23].

Rasch theory is based on the assumption that some items are harder and require more of the underlying trait than others and that some people have more of the latent trait than others, thereby, having a greater probability of responding positively to the more difficult items. Furthermore, items conform to a Guttman structure whereby they are ordered in terms of difficulty on a continuum. In other words, if a child has a certain level of developmental ability it is assumed that they ought to score positively for all items which require less difficulty than they possess [24].

A key underlying demand of the Rasch model is invariance [25] This means that the relative location of any two persons on the scale is independent of the items used and conversely the relative location of any two items on the continuum is independent of the person on which they are measured. The item and person locations are estimated separately but on the same scale. The separation of items and persons is a key advantage of Rasch modelling over CTT as it allows for generalisation across samples and items. Rasch modelling also provides a range of unique tools for testing the extent to which items and persons produce data that fit the Rasch model [25].

The EDI was not designed for use at the individual level but is used to detect change at the level of the school or the community. However, regardless of the purpose to which a tool is put it has to adhere to scientific measurement properties. The EDI can therefore benefit from Rasch analysis in that the extent to which each of the five scales meet the basic measurement properties outlined above can be examined. In particular, invariance, consistency of the interval levels and the hierarchy of competencies can be determined.

#### Data analysis

The data were analysed with the unidimensional Rasch model using RUMM2030 software [26]. The Rasch model was used to examine whether the EDI scales met the measurement requirements of invariance, allowing responses to be summated across items. In order to allow different numbers of categories and different threshold values across items the unconstrained (partial credit) Rasch model was applied.

Three aspects of the EDI were analysed: scale to sample targeting; overall scale fit to the Rasch model; and the extent to which individual items satisfied Rasch criteria.

### Scale to sample targeting

Person-item threshold distributions were examined to explore the relationship between the difficulty level of the items in each scale and the ability levels of those taking the test. These histograms, using the convention of Rasch analysis, are always centred at zero logits for the item location scale. Perfect targeting requires the item and person location means to both be zero.

#### Overall scale fit to the Rasch model

A number of tests were used to examine the extent to which each scale conformed to the Rasch model. Standardised mean and standard deviation (SD) values for item and person fit residuals are a way of representing the fit of both item and person data to the Rasch model. A mean value of zero with a SD of 1.0 would represent perfect fit (values less than 1.4 are considered acceptable for the SD). A further test examines the extent to which the hierarchical order of difficulty for items varies across class intervals of the measurement continuum. This is examined using a Chi-square statistic. A statistically significant Chi-square value (having performed a Bonferroni adjustment at the 0.05 probability level) indicates a problematic interaction between items and the latent trait being measured. A final test, known as the Person Separation Index (PSI) examines the extent to which the scale reliably discriminates between persons of different ability. The PSI can be produced with or without extreme values so that the extent of floor and ceiling effects on reliability can be examined. For scales which are intended to be used at the group level, a minimum PSI value of 0.7 is recommended.

#### Analysis of individual items

##### Threshold ordering

One of the requirements of the Rasch model is ‘category ordering’. This means that the hierarchical order of response options for particular items should accord with the latent variable in question. In other words, persons with higher levels of overall ability on a particular trait should be more likely than persons with lower ability to endorse item response options that are meant to capture higher levels of ability.

##### Item location

The location indicates the place on the continuum of difficulty where each item is located. Location is measured on the logit scale and lower scores represent lower levels of difficulty. The fit residuals provide an estimate of the extent to which the variance associated with each item is in accord with the Rasch model. The residuals shown are standardised and values between +/−2.5 demonstrate adequate fit. A test of item-trait interaction is also available. As with the test of overall scale fit, the Chi Square test is used to analyse whether items perform consistently across the continuum of difficulty. The test is Bonferroni adjusted at the 0.05 level and statistically significant values indicate problematic item-trait interaction.

##### Local response dependency

The Rasch model demands that responses to items on the same scale must be independent, that is, not conditional upon each other. For example, an item about spelling ability would be dependent on an item measuring ability to read implying that one of the items is redundant. Response dependency can be detected by examining the residual correlation between items after extraction of the Rasch model. Inter-item correlations greater than 0.4 are a strong signal for local response dependency.

##### Differential item functioning

One of the advantages of Rasch modelling is the possibility of detecting Differential Item Functioning (DIF). DIF occurs when different groups respond differently to an item despite having the same levels of the overall trait being measured. For example, if boys were to consistently score higher than girls on a particular item in an intelligence test, despite there being no gender differences in overall intelligence as measured by the scale, then DIF would be present in that item.

Every item was examined for DIF between male and female children in the sample. DIF was explored in RUMM through an analysis of variance (ANOVA) of the standardized response residuals for each item between genders. A Bonferroni adjusted p-value was then used to determine statistical significance. Item characteristic curves were examined to determine the direction of bias introduced in items where significant DIF was detected.

## Results

### Descriptive statistics

Data were available for 1344 children. Descriptive statistics for each scale are shown in Table 1. The mean and standard deviation (SD) for each scale is only provided for subjects with complete data on each scale (i.e. there has been no imputation). There was a strong positive skew on all five scales. There was also a marked ceiling effect on some scales with large numbers of children achieving the maximum possible score. This was most apparent for the communication skills and general knowledge scale where 34 % of children with complete items achieved the maximum score. The ceiling effect was least apparent for the emotional maturity scale (6 % of children with complete items achieved the maximum score).

**Table 1:** Descriptive statistics for each scale

Scale	Theoretical range	Mean (SD)	Min score *N*	Max score *N*	Item(s) missing *N*
Physical health and well-being	0–19	16.3 (3.1)	0	404	223
Social competence	0–52	42.5 (9.8)	0	235	90
Emotional maturity	0–60	45.7 (10.1)	0	68	261
Language and cognitive development	0–26	22.5 (4.7)	1	337	261
Communication & general knowledge	0–16	11.7 (4.7)	13	446	26

### Scale to sample targeting

**Fig. 1:**
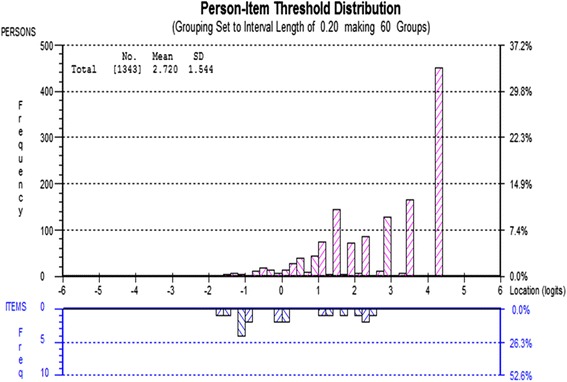
Person-item threshold distribution for the Physical Health and Well-being scale

For some scales the person-item histograms demonstrate a poor match between the difficulty levels of the items and the ability levels of those taking the test. In Fig. 1, the mean person location is 2.7 (SD = 1.5) for the physical health and well-being scale. The difficulty range for item locations (−1.63 to 1.23) is inconsistent with the ability range observed in the sample (−1.78 to 4.39). This implies that there is higher ability in the sample than the difficulty levels measured by the items on the physical health and well-being scale and suggests that additional items at the higher levels of difficulty are required.

The social competence scale also demonstrate a mismatch between persons and items. The mean person location on the logit scale is 2.7 (SD = 2.0) and the difficulty range for item locations (−1.50 to 1.26) is inconsistent with the ability range observed in the sample (−3.72 to 5.47). This suggests a need for additional items at both the lower and higher ranges of difficulty.

**Fig. 2:**
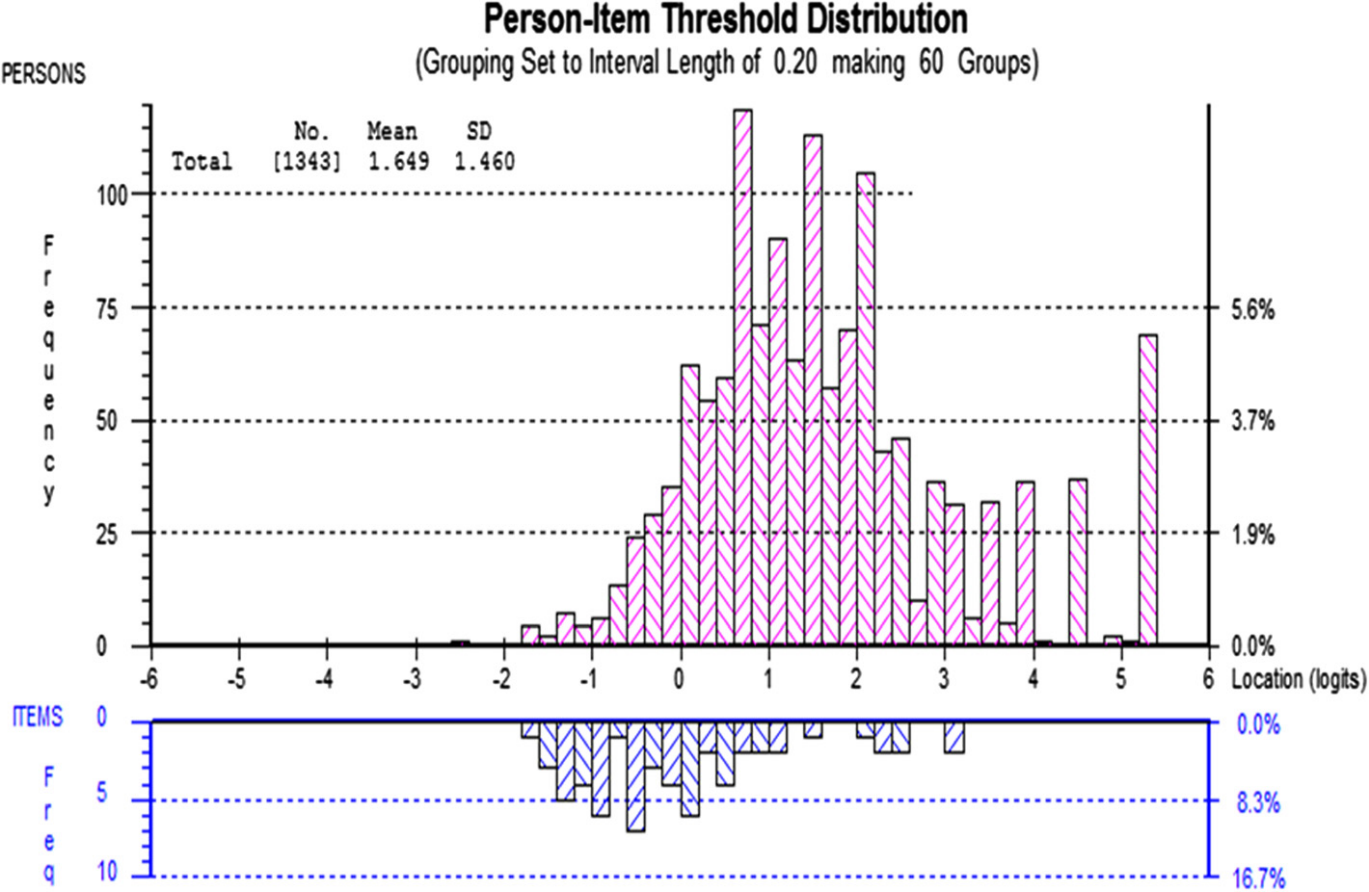
Person-item threshold distribution for the Emotional Maturity scale

In Fig. 2, the emotional maturity scale demonstrates a better match between sample and items. The highest levels of ability are still not addressed by the item set but this covers a smaller group of children. The mean person location is 1.6 on the logit scale (SD = 1.5) and the difficulty range for item locations (−1.27 to 1.99) is a better match with the ability range observed in the sample (−2.52 to 5.27).

Items on the language and cognitive development scale cover a very wide range of difficulty. The mean person location on the logit scale is 3.3 (SD = 2.1) and the difficulty range for item locations (−3.86 to 4.86) is a good match with the ability range observed in the sample (−4.99 to 5.86) but is still not enough to cover the highest levels of ability in the sample.

There is a poor match between persons and items on the communication and general knowledge scale. The mean person location on the logit scale is 1.9 (SD = 2.5) and the difficulty range for item locations (−1.11 to 1.03) is a poor match with the ability range observed in the sample (−4.46 to 4.39).

### Overall fit to the Rasch model

Table 2 displays summary Rasch model statistics for the five scales. These give an overall analysis of the extent to which the EDI successfully measures the sample according to the Rasch model paradigm.

**Table 2:** Summary of EDI scale fit to the Rasch model

Scale	Item residual Mean (SD)	Person residual Mean (SD)	Chi square Value	*p*	PSI with extremes	PSI without extremes
Physical health and well-being	−1.28 (5.51)	−0.39 (1.00)	813.82	<0.001	0.62	0.65
Social competence	−1.46 (3.53)	−0.43 (1.46)	658.53	<0.001	0.87	0.90
Emotional maturity	−0.87 (4.19)	−0.43 (1.33)	1678.47	<0.001	0.88	0.88
Language and cognitive development	−1.86 (1.76)	−0.41 (0.57)	382.94	<0.001	0.72	0.78
Communication skills and general knowledge	−1.78 (5.57)	−0.47 (1.31)	372.98	<0.001	0.83	0.85

All five EDI scales demonstrate problematic fit to the Rasch model. For all scales, item residual standard deviations are larger than 1.4. and there is evidence of statistically significant item-trait interaction in all scales, signalling some room for improvement in the content of each scale. On the other hand, all scales apart from physical health and well-being demonstrate an ability to reliably discriminate between persons of different ability as measured by the PSI.

In a separate analysis it is possible to identify the number of persons within the sample who fit the Rasch model. This gives a sense of the extent to which each scale has adequately measured the sample. The physical health and well-being scale performed very poorly on this metric with 452 persons (33.6 %) providing extreme standardised person-fit residuals (defined as outside the +/−2.5 range). The social competence scale fared better with 240 persons (17.9 %) providing extreme person-fit residuals. The emotional maturity scale had 72 persons (5.4 %) with extreme person-fit residuals. A high proportion of the sample (*N* = 409, 30.4 %) had extreme person-fit residuals on the language and cognitive development scale. 464 persons (34.5 %) had extreme person-fit residuals on the communication and general knowledge scale, the highest of all five scales.

### Analysis of individual items

#### Threshold ordering

Only one EDI item (‘sucks finger’ on the physical health and well-being scale) showed threshold disordering indicating that the response options for all but one item are performing as expected.

#### Item location

Table 3 shows the ordered item locations, fit residuals and probabilities for the physical health and well-being scale. Item 6 (‘established hand preference’) is the easiest item on the scale and item 11 (‘level of energy’) is the hardest item.

**Table 3:** Ordered item locations, fit residuals and probabilities for the physical health and well-being scale

Item	Item description	Location	SE	Fit residual	Chi square	Probability
6	established hand preference	−1.63	0.16	−1.10	7.74	0.356
5	independent in washroom	−1.57	0.15	−0.08	9.04	0.250
4	hungry	−1.15	0.14	1.61	13.87	0.054
1	over or underdressed	−1.04	0.13	1.09	13.84	0.054
7	well co-ordinated	0.00	0.10	−1.84	***46.23***	***0.000***
2	too tired or sick	0.04	0.10	0.61	***21.73***	***0.003***
13	sucks finger	0.23	0.07	**4.09**	***141.62***	***0.000***
10	climb stairs	0.37	0.07	**−7.26**	***74.23***	***0.000***
12	overall physical development	0.57	0.07	**−8.90**	***89.37***	***0.000***
9	manipulate objects	0.67	0.07	**−8.60**	***77.55***	***0.000***
3	late	1.13	0.08	**11.16**	***292.69***	***0.000***
8	proficiency with pen	1.15	0.06	**−4.66**	15.69	0.028
11	level of energy	1.23	0.06	**−2.83**	10.22	0.176

With respect to individual item fit, items 13 through 11 all fail the fit residual test and items 7 through 3 all fail the Chi square test for item-trait interaction (Bonferroni adjusted *p* values <0.003846) - as outlined in bold on the table.

Table 4 shows the ordered item locations, fit residuals and probabilities for the social competence scale. Item 19 (‘play with new toy’) is the easiest item on the scale and item 1 (‘overall social/emotional development’) is the hardest item. Fourteen items (9, 16, 6, 23, 10, 5, 3, 13, 7, 24, 15, 26, 8, 12) demonstrate extreme fit residuals and ten items (19, 9, 16, 6, 5, 18, 3, 13, 26, 8) fail the Chi square test for item-trait interaction (Bonferroni adjusted *p* values <0.001923).

**Table 4:** Ordered item locations, fit residuals and probabilities for the social competence scale

Item	Item description	Location	SE	Fit residual	Chi square	Probability
19	play with new toy	−1.50	0.09	0.55	***27.84***	***0.001***
20	play a new game	−1.29	0.08	−0.90	19.38	0.013
9	respect for adults	−1.08	0.08	**−4.11**	***30.39***	***0.000***
16	takes care of school materials	−0.85	0.08	**−4.63**	***34.62***	***0.000***
6	respects others property	−0.82	0.08	**−3.71**	***25.42***	***0.001***
21	play with new book	−0.82	0.08	0.46	18.29	0.019
23	follow one-step instructions	−0.78	0.07	**−4.11**	23.09	0.003
10	respect for children	−0.72	0.07	**−2.86**	19.03	0.015
5	follow rules and instructions	−0.29	0.07	**−6.36**	***43.16***	***0.000***
18	curious about world	−0.19	0.07	2.35	***25.37***	***0.001***
3	plays and works with other	−0.11	0.07	**−5.33**	***34.16***	***0.000***
25	adjust to change in routines	−0.05	0.07	−0.97	7.80	0.453
13	follows directions	0.05	0.07	**−7.85**	***62.29***	***0.000***
7	self-control	0.11	0.07	**−3.86**	15.66	0.047
4	play with various children	0.34	0.06	0.27	10.90	0.207
24	follow class routines	0.37	0.06	**−3.42**	17.56	0.025
11	responsibility for actions	0.39	0.06	−2.01	10.41	0.237
15	works independently	0.56	0.06	**−3.18**	12.53	0.129
22	solve day-to-day problems	0.59	0.06	−0.77	8.46	0.390
26	tolerance of mistakes	0.60	0.06	**7.63**	***89.84***	***0.000***
8	self-confidence	0.71	0.06	**5.78**	***58.82***	***0.000***
17	works neatly	0.76	0.06	1.48	8.90	0.351
14	completes work on time	0.87	0.06	1.56	12.76	0.121
2	get along with peers	0.92	0.06	−0.69	11.69	0.165
12	listens attentively	0.96	0.06	**−3.71**	22.14	0.005
1	overall social/emotional dev	1.26	0.06	0.43	8.03	0.431

Table 5 shows the ordered item locations, fit residuals and probabilities for the emotional maturity scale. Item 13 (‘takes things’) is the easiest item on the scale and item 3 (‘stop a quarrel’) is the hardest item. Sixteen items (12, 19, 26, 18, 27, 21, 22, 9, 20, 15, 16, 23, 1, 30, 8, 4) demonstrate extreme fit residuals and 19 items (12, 19, 26, 18, 27, 21, 22, 9, 20, 16, 23, 1, 17, 30, 5, 8, 6, 4, 7) fail the Chi square test for item-trait interaction (Bonferroni adjusted *p* values <0.001667).

**Table 5:** Ordered item locations, fit residuals and probabilities for the emotional maturity scale

Item	Item description	Location	SE	Fit residual	Chi square	Probability
13	takes things	−1.27	0.08	−2.35	17.80	0.038
12	kicks bites hits	−1.15	0.07	**−3.42**	***28.64***	***0.001***
24	unhappy, sad, depressed	−1.02	0.07	−0.76	16.38	0.059
14	laughs at discomfort	−0.98	0.07	−0.24	15.98	0.067
10	physical fights	−0.97	0.07	−1.87	19.20	0.024
11	bullies others	−0.96	0.07	−2.46	13.10	0.158
19	temper tantrums	−0.89	0.07	**−4.01**	***37.55***	***0.000***
25	fearful or anxious	−0.80	0.06	0.61	17.29	0.044
29	incapable of making decisions	−0.65	0.06	−0.86	8.45	0.490
26	worried	−0.64	0.06	**2.89**	***40.12***	***0.000***
18	disobedient	−0.61	0.06	**−2.97**	***48.74***	***0.000***
27	cries a lot	−0.60	0.06	**2.66**	***33.87***	***0.000***
28	nervous, tense	−0.50	0.06	0.29	15.72	0.073
21	difficulty awaiting turn	−0.41	0.06	**−2.81**	***33.48***	***0.000***
22	can’t settle to anything	−0.39	0.06	**−4.40**	***55.02***	***0.000***
9	upset when left	−0.16	0.05	**10.72**	***337.63***	***0.000***
20	impulsive	−0.04	0.05	**−3.90**	***39.87***	***0.000***
15	restless	0.05	0.05	**−3.09**	24.28	0.004
16	distractible	0.23	0.05	**−2.97**	***42.87***	***0.000***
23	is inattentive	0.24	0.05	**−3.14**	***53.95***	***0.000***
1	help someone hurt	0.28	0.05	**−3.52**	***44.51***	***0.000***
17	fidgets	0.32	0.05	−1.50	***31.07***	***0.000***
30	shy	0.41	0.05	**15.02**	***507.74***	***0.000***
5	comfort a crying child	1.14	0.05	−2.23	***35.26***	***0.000***
2	clear up a mess	1.23	0.05	−1.43	10.72	0.295
8	help sick children	1.39	0.05	**−2.83**	***33.12***	***0.000***
6	picks up objects	1.39	0.05	0.09	***41.64***	***0.000***
4	help other children	1.47	0.05	**−4.09**	***30.30***	***0.000***
7	invite bystanders to join	1.87	0.05	−1.63	***27.46***	***0.001***
3	stop a quarrel	1.99	0.05	−1.89	16.70	0.054

Table 6 shows the ordered item locations, fit residuals and probabilities for the language and cognitive development scale. Item 1 (‘handle a book’) is the easiest item on the scale and item 9 (‘read complex words’) is the hardest item. Nine items (3, 6, 8, 10, 15, 17, 18, 21, 24) demonstrate extreme fit residuals and six items (6, 8, 9, 10, 11, 15) fail the Chi square test for item-trait interaction (Bonferroni adjusted *p* values <0.001923).

**Table 6:** Ordered item locations, fit residuals and probabilities for the language and cognitive development domain

Item	Item description	Location	SE	Fit residual	Chi square	Probability
1	handle a book	−3.86	0.35	−1.34	3.12	0.874
20	sort by common characteristics	−2.25	0.19	−0.40	3.50	0.835
21	use one-to-one correspondence	−1.71	0.16	**−2.53**	9.77	0.202
2	interested in books	−1.64	0.16	−2.29	5.40	0.611
25	recognise shapes	−1.21	0.14	0.54	7.89	0.343
19	interested in number games	−0.81	0.13	−0.34	8.43	0.296
18	interested in maths	−0.78	0.13	**−3.95**	19.67	0.006
5	attach sounds to letters	−0.63	0.12	−1.41	4.35	0.738
4	identify 10 letters	−0.62	0.12	−2.48	7.59	0.370
12	aware of writing direction	−0.62	0.12	−1.30	5.04	0.655
11	experiment with writing	−0.62	0.12	1.23	***34.05***	***0.000***
14	writing his/her name	−0.50	0.12	−1.51	9.32	0.231
3	interested in reading	−0.45	0.12	**−3.29**	9.13	0.243
26	understands time	−0.40	0.12	−0.54	7.14	0.414
24	say which is bigger than 2	−0.39	0.12	**−2.63**	7.65	0.364
7	group reading activities	0.06	0.11	−1.64	15.09	0.035
8	read simple words	0.24	0.10	**−5.14**	***29.66***	***0.000***
17	remember things easily	0.74	0.09	**−2.53**	8.60	0.282
23	recognise 1–10	0.77	0.09	−1.96	13.00	0.072
15	write simple words	0.84	0.09	**−4.97**	***30.98***	***0.000***
6	awareness of rhyming	0.98	0.09	**−3.01**	***23.35***	***0.001***
10	read simple sentences	1.58	0.09	**−5.67**	***38.08***	***0.000***
22	count to 20	1.95	0.08	−0.07	13.50	0.061
13	writing voluntarily	1.97	0.08	−1.02	17.88	0.013
16	write simple sentences	2.51	0.08	−0.09	22.20	0.002
9	read complex words	4.86	0.10	−0.04	***28.56***	***0.000***

Table 7 shows the ordered item locations, fit residuals and probabilities for the communication and general knowledge scale. Item 1 (‘handle a book’) is the easiest item on the scale and item 9 (‘read complex words’) is the hardest item. Six items (8, 6, 5, 4, 1, 3) demonstrate extreme fit residuals and fail the Chi square test for item-trait interaction (Bonferroni adjusted *p* values <0.006250).

**Table 7:** Ordered item locations, fit residuals and probabilities for the communication skills and general knowledge scale

Item	Item description	Location	SE	Fit residual	Chi square	Probability
8	knowledge of world	−1.11	0.08	**7.19**	***101.05***	***0.000***
2	ability to listen	−0.47	0.07	−0.16	21.08	0.007
6	understand what is being said	−0.44	0.07	**−5.06**	***46.85***	***0.000***
5	communicate needs	0.09	0.07	**−6.26**	***36.25***	***0.000***
4	imaginative play	0.20	0.07	**5.33**	***53.36***	***0.000***
7	articulate clearly	0.31	0.07	−1.48	8.44	0.391
1	ability to use English	0.37	0.07	**−6.96**	***40.65***	***0.000***
3	ability to tell story	1.03	0.07	**−6.87**	***65.31***	***0.000***

#### Local response dependency

Only one instance of local response dependency was observed for the physical health and well-being scale, between item 8 (‘proficiency with pen’) and item 9 (‘manipulate objects’). The items are very close conceptually and have an intuitive causal relationship.

Four instances of local response dependency were observed for the social competence scale. These were items 1 and 2 (‘overall social/emotional development and ‘get along with peers’), items 3 and 4 (‘plays and works with others’ and ‘plays with various children’), items 9 and 10 (‘respect for adults’ and ‘respect for children’) and items 14 and 15 (‘completes work on time’ and ‘works independently’).

Twenty-three item-pairs demonstrated local response dependency on the emotional maturity scale which suggests a problem with many item relationships. The pairs were: 1–5, 1–8, 2–6, 3–4, 3–5, 3–8, 4–5, 4–8, 5–8, 7–8, 10–12, 11–12, 15–16, 15–17, 15–20, 15–22, 16–17, 16–22, 16–23, 17–23, 22–23, 25–26, 25–28.

There was only one instance of local response dependency in the language and cognitive development scale. This was between item 2 (‘interested in books’) and item 3 (‘interested in reading’). The items are very close conceptually and have an intuitive causal relationship.

There were no instances of local response dependency on the communication skills and general knowledge scale.

### Differential item functioning

DIF for gender is evident for two items on the physical health and well-being scale. Item 3 (‘late’; F = 18.03) and item 9 (‘manipulates objects’; F = 12.28) displayed significant DIF by gender (Bonferroni adjusted *p* values <0.001282). Analysis of the item characteristic curves revealed that at equivalent levels of physical health and well-being boys were more likely than expected to be rated positively on item 3 (i.e. to not be late), whereas girls were more likely than expected to be rated positively on item 9 (i.e. to be able to manipulate objects).

**Fig. 3:**
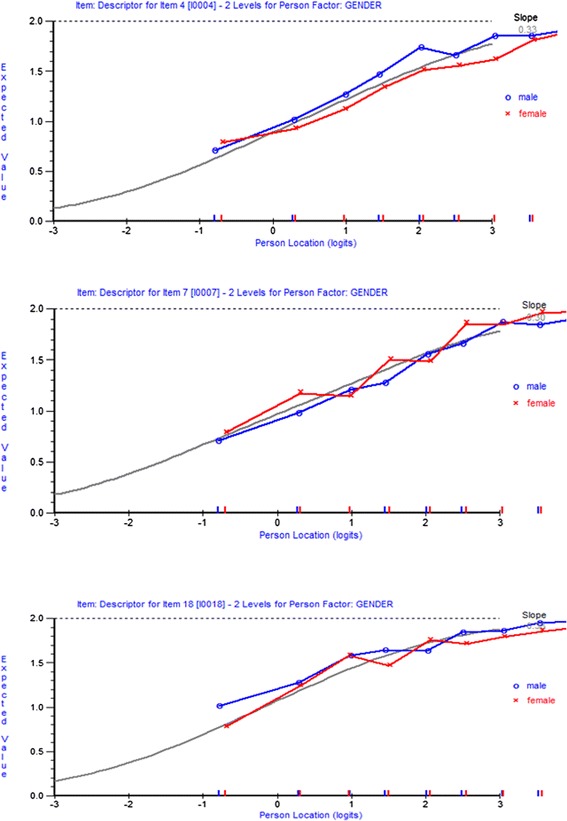
Gender DIF for social competence scale (items 4, 7 and 18)

DIF for gender on the social competence scale is outlined in Fig. 3. Item 4 (‘play with various children’; F = 13.65), item 7 (‘self-control; F = 14.17) and item 18 (‘curious about world’; F = 16.24) displayed significant DIF by gender (Bonferroni adjusted *p* values <0.000641). At equivalent levels of social competence boys were more likely than expected to be rated as able to play with various children, girls were more likely than expected to be rated as having self-control, and boys were more likely than expected to be rated as being curious about the world.

Eleven items on the emotional maturity scale showed significant DIF by gender (Bonferroni adjusted *p* values <0.000556). These were item 1 (‘help someone hurt’; F = 13.73), item 5 (‘comfort a crying child’; F = 15.24), item 6 (‘picks up objects’; F = 23.18), item 10 (‘physical fights’; F = 16.85), item 12 (‘kicks, bites, hits’; F = 17.64), item 15 (‘restless’; F = 14.95), item 17 (‘fidgets’; F = 13.73), item 18 (‘disobedient’; F = 11.97), item 20 (‘impulsive’; F = 12.88), item 22 (‘can’t settle to anything’; F = 13.87) and item 30 (‘shy’; F = 58.76). Most of this item bias favoured girls. At equivalent levels of emotional maturity, girls were more likely than boys to be rated as likely to help someone hurt, comfort a crying child, avoid physical fights, not kick/bite/hit, not be restless, not fidget, be obedient, not be impulsive, and to be able to settle. On two items (likely to pick up objects and likely to not be shy) the direction of bias favoured boys.

DIF for gender was evident for only one item on the language and cognitive scale. Item 23 (‘recognise 1–10’; F = 13.50) showed significant DIF by gender (Bonferroni adjusted *p* value <0.000641). At equivalent levels of language and cognitive development boys were more likely than expected to be rated as able to recognise numbers between 1 and 10. No significant DIF by gender was present for any item on the communication skills and general knowledge scale.

### Summary of findings in relation to each scale

The findings in relation to each scale can be summarised as follows:

#### Physical health and well being (13 items)

The scale did not discriminate well between children of differing ability and showed evidence of item-trait interaction. In total 33.6 % of children showed extreme person fit residuals. There was a mismatch between ability and item difficulty with additional items needed at the upper end of the scale. One item showed disordered thresholds. Seven items had extreme fit residuals and seven showed item-trait interaction. One local response dependency between items was observed. Two items displayed DIF by gender with one showing item bias favouring girls and the other favouring boys.

#### Social competence (26 items)

The social competence scale reliably discriminated between children of different abilities. However, there was evidence of item-trait interaction at the scale level and 17.9 % of children showed extreme fit residuals. There were similar levels of person-item mismatch to the physical health and well-being scale. Fourteen items had extreme fit residuals and ten showed item-trait interaction. Four instances of local response dependency between items were observed. Three items displayed DIF by gender with two showing item bias favouring boys and one favouring girls.

#### Emotional maturity (30 items)

The emotional maturity scale reliably discriminated between children of differing abilities and had item residuals close to zero. Only 5.4 % of children had extreme fit residuals. This scale had the best match between persons and items. However, 16 items had extreme fit residuals and 19 showed item-trait interaction. Twenty-three instances of local response dependency between items were observed. Eleven items showed DIF by gender with nine showing item bias favouring girls and two favouring boys.

#### Language and cognitive development (26 items)

The language and cognitive development scale was capable of reliably discriminating between persons of differing ability but again, there was evidence of item trait interaction and 30.4 % of children had extreme fit residuals. This scale covered a wide range of difficulty but still not enough to cover the upper range of ability. Nine items demonstrated extreme fit residuals and six items showed item-trait interaction. One instance of local response dependency between items was observed and one item displayed DIF by gender with the bias favouring boys.

#### Communication skills and general knowledge (8 items)

The communication skills and general knowledge scale discriminated between children of differing ability, but did show item-trait interaction. The percentage of children with extreme fit residuals was 34.5 %. The ceiling effect, which was apparent across all scales, was most marked for this domain. Six items demonstrated extreme fit residuals and six showed item-trait interaction. There was no instance of local response dependency between items and no DIF by gender.

## Discussion

This paper used Rasch analysis to explore the psychometric properties of the five domains of the EDI in a sample of 1344 children in Ireland. The aim of the study was to determine the psychometric properties of the EDI within the Rasch paradigm.

Every scale demonstrated some elements which are of concern. However, the Rasch criteria are very demanding and they have to be taken as a whole Pallant and Tennant [22]. No one criterion is disqualifying.

All scales had an inadequate number of items for measuring ability at the higher levels with a marked ceiling effect. Similar patterns were observed in the Australian and Swedish Rasch analysis of the EDI [19, 20]. In the Australian study, Andrich and Styles [19] took the view that, as the instrument was developed for the explicit purpose of identifying children at risk (at the lower end of the spectrum), it was not necessary to discriminate between children who were performing above this level. However, the ceiling effects observed in this study create three important problems that persist regardless of the focus of the instrument. First, it has implications for the use of an arbitrary cut-off point of 10 %. If the domain in question has a large ceiling effect it implies that children with high absolute scores may be classified as relatively ‘at risk’. In other words, the standard for what constitutes ‘at risk’ becomes higher and there is the danger that children who would be considered within the normal spectrum of development on other measures are classified as at risk on the EDI. The EDI would eventually become synonymous with over-diagnosis in such a scenario. One way to address this problem is to use Rasch Modelling to set the cut points so that they take account of both the distribution of score and the hierarchy of competencies. Second, the ceiling effect is problematic for studies that aim to use the EDI to compare populations as it will lead to an underestimate of the difference between geographical areas with high and low levels of developmental deprivation. Third, the EDI is used extensively to measure changes over time resulting from early childhood interventions. It is essential, therefore, that the full range of possible improvements at the domain level can be detected.

The concept of healthy child development, which underpins the EDI, needs to be fully articulated at all levels of ability. Hobart et al. [27] outline the need for a bottom-up approach to instrument development which would begin with a construct theory onto which items would be mapped using both qualitative and quantitative methods. This approach could serve well as a detailed evaluation of the EDI.

The DIF for gender, which is particularly evident in the emotional maturity scale, also needs attention. For the most part, DIF for gender is not unexpected and can achieve a balance between items that favour girls and boys. However, in this instance, almost one-third (9 out of 30) items on this scale are biased in favour of girls meaning that despite having the same overall levels of emotional maturity as boys, girls score better than expected on these items. Gendered differences in emotional and social expression are evident from an early age [28] and need to be addressed in the context of the measurement of early childhood development.

The nine items which were biased in favour of girls were primarily associated with pro-social behaviours and inattentive behaviours. Girls were more likely than boys (at the same level of emotional maturity) to be rated as likely to help someone hurt, comfort a crying child, avoid physical fights, not kick/bite/hit, not be restless, not fidget, be obedient, not be impulsive, and to be able to settle. This may be indicative that there are certain areas where boys and girls express their emotional immaturity in different ways and that the EDI is picking this up. However, it may reflect gender pre-conceptions among teachers. Further qualitative research is needed to explore this.

The emotional maturity scale requires attention, particularly at the level of the individual items. It is the longest scale consisting of 30 items. In addition to DIF, 23 pairs of local response dependency were observed. Item 5 (comforts a crying child), item 3 (helps someone hurt), item 4 (helps other children) and item 8 (helps sick children) all interact with each other. Moreover, items 3 and 5 showed gender DIF favouring girls. All of these items are indicators of helping behaviour. Another group of items which show a marked degree of response dependency are item 15 (restless), item 16 (distractible), item 17 (fidgets), item 20 (impulsive) and item 22 (can’t settle). Again, items 15, 17, 20 and 22 showed DIF favouring girls. These are two instances where the instrument may benefit from qualitative work with teachers and others in the field of education with a view to item reduction.

In order to improve the EDI scales a range of options need to be considered. First, qualitative work to explore how various items are rated would be useful. This would deepen our understanding of issues such as the causes of the high level of DIF displayed by the emotional maturity domain. Second, delete problematic items to determine whether or not the EDI scales can be made to better fit the Rasch model. This quantitative approach should only be performed in conjunction with qualitative research however, as it is just as important to understand the source of misfit as it is to eliminate it. Third, performing qualitative and quantitative research to produce additional items to fill obvious gaps would be particularly useful for the higher levels of ability on all the scales.

The findings highlight the value of Rasch analysis in the psychometric evaluation of rating scales. The EDI had demonstrated sound psychometric properties when evaluated using traditional psychometric tests. However, traditional methods are concerned with total scores on scales. As a result, poorly functioning individual items can remain undetected [25]. This study has allowed a detailed examination of the items which make up the five scales of the EDI.

### Limitations

The Rasch analysis outlined above is the first step in a process of refining the EDI for use in the Irish context. It did not involve any adjustment to the instrument. Further qualitative and quantitative research will be required to test the impact of removing or adding items to the scales.

The authors approached the implementation of the EDI in Ireland from a population-health perspective and the need for an instrument which could identify populations or communities of children at risk, thereby informing policy and services supporting early childhood development. In this context it was essential that we examine the psychometric properties of the EDI. We have identified a number of areas of concern but will not make adjustments to the instrument without detailed consultation with specialists in early education and particularly with Professor Janus of the Offord Centre who developed the instrument and who has been involved with its international adaptation. This level of work was beyond the scope of this study.

## Conclusion

The study points to a number of problems with the EDI which should be addressed in further research. If the EDI is to be implemented at a national level in Ireland, it would benefit from further refinement which could in turn inform the international implementation of the EDI.

## Abbreviations

AEDC: Australian early development census
AEDI: Australian early development index
ANOVA: analysis of variance
CSGK: communication skills and general knowledge
CTT: classical test theory
DIF: differential item functioning
EDI: early development instrument
EM: emotional maturity
LCD: language and cognitive development
LSAC: longitudinal study of Australian children
PHWB: physical health and well-being
PPVT: peabody picture vocabulary test
PSI: person separation index
SC: social competence
SD: standard deviation
STROBE: strengthening the reporting of observational studies in epidemiology
